# Genetic dissection of resistance to gray leaf spot by genome-wide association study in a multi-parent maize population

**DOI:** 10.1186/s12870-023-04701-1

**Published:** 2024-01-02

**Authors:** Can Hu, Tianhui Kuang, Ranjan K. Shaw, Yudong Zhang, Jun Fan, Yaqi Bi, Fuyan Jiang, Ruijia Guo, Xingming Fan

**Affiliations:** 1 Institute of Food Crops, Yunnan Academy of Agricultural Sciences, Kunming, China; 2 School of Agriculture, Yunnan University, Kunming, China

**Keywords:** Maize, Gray leaf spot, SNP, Haplotype, GWAS

## Abstract

**Background:**

Understanding the genetic mechanisms underlying gray leaf spot (GLS) resistance in maize is crucial for breeding GLS-resistant inbred lines and commercial hybrids. Genome-wide association studies (GWAS) and gene functional annotation are valuable methods for identifying potential SNPs (single nucleotide polymorphism) and candidate genes associated with GLS resistance in maize.

**Results:**

In this study, a total of 757 lines from five recombinant inbred line (RIL) populations of maize at the F_7_ generation were used to construct an association mapping panel. SNPs obtained through genotyping-by-sequencing (GBS) were used to perform GWAS for GLS resistance using a linear mixture model in GEMMA. Candidate gene screening was performed by analyzing the 10 kb region upstream and downstream of the significantly associated SNPs linked to GLS resistance. Through GWAS analysis of multi-location phenotypic data, we identified ten candidate genes that were consistently detected in two locations or from one location along with best linear unbiased estimates (BLUE). One of these candidate genes, *Zm00001d003257* that might impact GLS resistance by regulating gibberellin content, was further identified through haplotype-based association analysis, candidate gene expression analysis, and previous reports.

**Conclusions:**

The discovery of the novel candidate gene provides valuable genomic resources for elucidating the genetic mechanisms underlying GLS resistance in maize. Additionally, these findings will contribute to the development of new genetic resources by utilizing molecular markers to facilitate the genetic improvement and breeding of maize for GLS resistance.

**Supplementary Information:**

The online version contains supplementary material available at 10.1186/s12870-023-04701-1.

## Introduction

Maize gray leaf spot (GLS) is a severe foliar disease that poses a significant threat to maize production and yield [1]. Maize GLS was initially reported in the United States [2], and has since become endemic in several countries, including Brazil, Uganda, South Africa and other countries of South America and Africa [3–7]. In China, GLS was first reported in 1991 [8]. Maize GLS resistance is a complex quantitative trait controlled by multiple genes. Numerous studies have demonstrated the high heritability of GLS and revealed that the quantitative trait loci (QTL) associated with maize GLS resistance exhibit mainly additive effects, with some displaying minor dominant effects [3, 9]. Studies have revealed that the causal agents of GLS are primarily two main fungus species, *Cercospora zeae-maydis* and *Cercospora zeina*, responsible for causing GLS in maize worldwide [10]. However, there are regional variations in the causal fungus species. For instance, in the United States and Brazil, at least two fungus species are present [4, 11], while in Africa, it is primarily *Cercospora Zeina* [12]. In Northern China, GLS is primarily caused by the fungus *Cercospora zeae-maydis*, whereas in Southwest China and other regions like Yunnan, it is predominantly caused by *Cercospora zeina*. The economic losses inflicted by GLS on maize yield are substantial, and studies have shown that developing resistant varieties is the most effective approach to combat GLS [11]. Therefore, the identification of candidate genes associated with GLS resistance, unravelling the genetic basis of GLS resistance in maize, and subsequent breeding of resistant maize varieties are of utmost importance in sustaining maize yields and meeting the food demands of the growing global population.

Due to advancements in sequencing technology and analytical methods, QTL mapping [13] and GWAS [14] have emerged as important approaches in discovering candidate genes related to maize GLS resistance. Researchers have successfully identified numerous QTLs and SNPs associated with maize GLS resistance by employing diverse varieties and lines from maize populations through QTL mapping and GWAS. These studies have laid a crucial foundation for elucidating the genetic mechanisms underlying GLS resistance in maize. However, these studies have rarely utilized tropical or subtropical maize germplasm as the analyzed population, despite the importance of tropical and subtropical germplasms in combatting maize diseases. In our study, we selected one subtropical and four tropical GLS-resistant RILs as the female parents and one temperate GLS-susceptibility RIL as the common male parent. The objectives of the study were to (1) exploit tropical and subtropical maize germplasm resources to uncover important genetic loci and candidate genes regulating GLS resistance; (2) lay the foundation for fine mapping and cloning of GLS resistance genes in maize; and (3) provide a theoretical basis for genetic improvement of GLS resistance in maize.

## Results

### Maize GLS levels based on phenotypic data

In this study, a descriptive statistical analysis was performed to assess the infection level of GLS in five RIL populations at two different locations. The results showed that the average infection level of GLS in the plants from the five RIL populations in Dehong (DH) and Baoshan (BS) ranged from 3 to 6 (Table 1). Meanwhile, the absolute values of skewness and kurtosis coefficients for the five populations in both locations were close to 1, indicating that the GLS levels in the test populations at both locations followed a normal distribution and exhibited typical quantitative trait characteristics. GWAS was performed based on this phenotypic data.

**Table 1:** Statistical analysis of GLS phenotypes of the five RIL populations in DH and BS

Location	Population	Mean	Standard Deviation (SD)	Skewness	kurtosis	Coefficient of Variation (CV)(%)
DH	RIL-CML312	3.490	1.357	0.275	-0.373	38.879
	RIL-YML32	6.181	1.132	-1.222	1.764	18.321
	RIL-YML16	5.548	1.295	-0.718	0.08	23.346
	RIL-YML226	5.378	1.181	-1.163	1.578	21.961
	RIL-D39	5.936	1.076	-0.972	1.097	18.125
BS	RIL-CML312	3.927	1.272	-0.025	-0.529	32.386
	RIL-YML32	5.502	1.360	-0.709	1.406	24.720
	RIL-YML16	3.610	1.568	0.434	-0.055	43.450
	RIL-YML226	3.822	1.777	-0.109	-1.075	46.491
	RIL-D39	3.674	1.830	0.203	-0.714	49.821

### Genotyping-by-sequencing and SNP data profile

We employed the GBS approach to sequence 757 RILs. After filtering, each RIL had an average of 3.78 Gb clean reads with a depth of 12.68X and a coverage of 12.12%. On average, the alignment rate of the samples was 98.56%, and the coverage of at least four bases was 4.91%. These results indicated that the sequencing coverage for each sample was sufficient to adequately cover the reference genome, meeting the requirements for re-sequencing analysis.

### Principal component analysis, population structure and kinship analysis

**Fig. 1:**
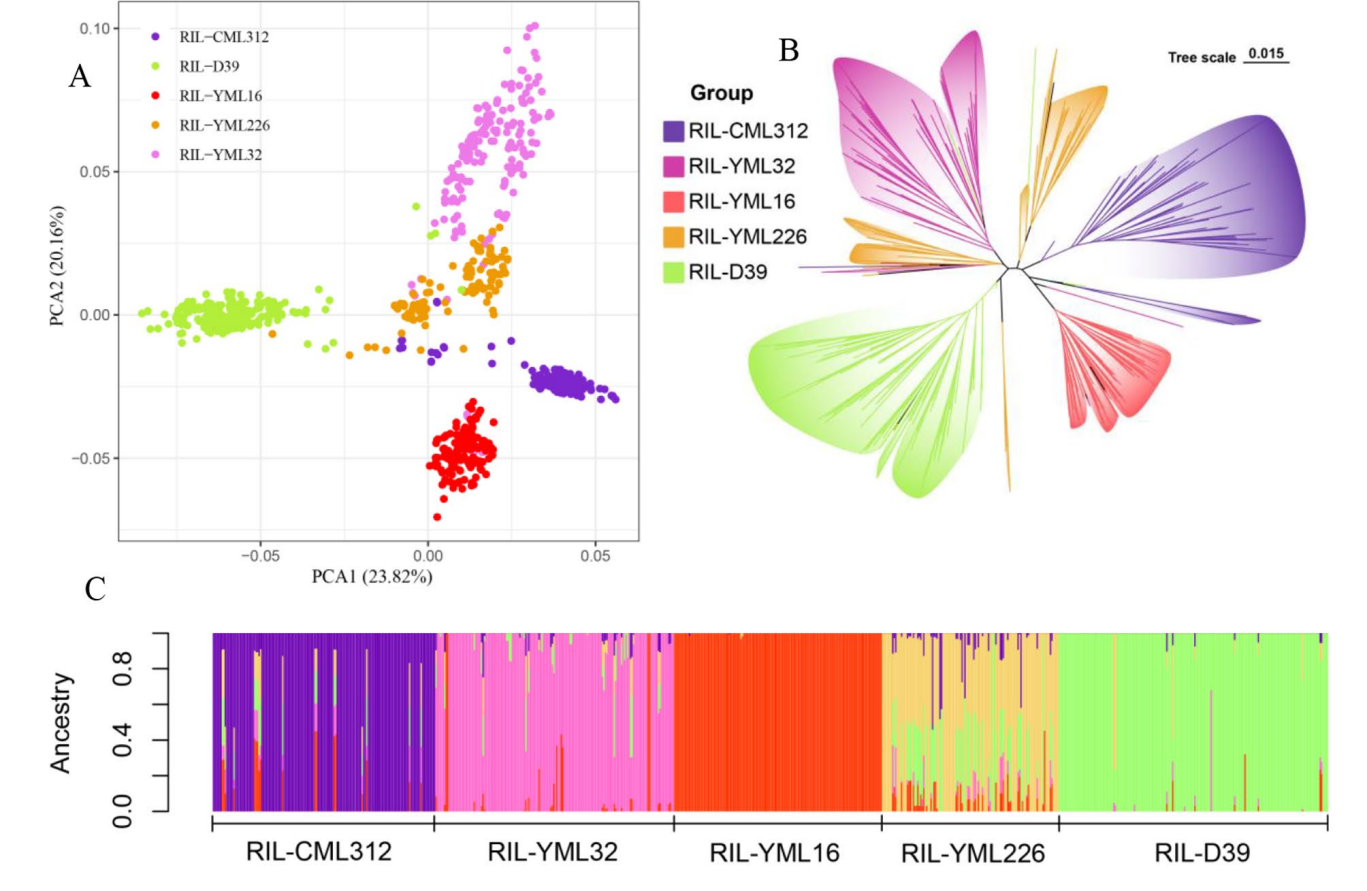
The population structure of 757 recombinant inbred lines (RILs). (**A**) Principal component analysis shows the clustering of RILs, with purple, pink, red, yellow, and green dots representing the RIL-CML312, RIL-YML32, RIL-YML16, RIL-YML226, and RIL-D39 population, respectively. (**B**) The evolutionary tree with purple, pink, red, yellow, and green branches represent RIL-CML312, RIL-YML32, RIL-YML16, RIL-YML226, and RIL-D39, respectively. (**C**) Ancestral component analysis, where purple, pink, red, yellow, and green bars represent RIL-CML312, RIL-YML32, RIL-YML16, RIL-YML226, and RIL-D39, respectively

The results of the principal component analysis (PCA) showed that 757 maize RILs could be classified into five main groups based on PCA1 and PCA2 (Fig. 1A). We performed evolutionary tree construction based on the filtered SNP dataset, and the results showed that 757 maize RILs could be clustered into five major clusters (Fig. 1B). The ancestral component analysis was consistent with the results of PCA and evolutionary tree, and the population structure was clear and realistic at K = 5 (i.e., grouped into five clusters) (Fig. 1C). Therefore, we selected the first three PCAs as covariates to be included in the model of GWAS analysis along with the kinship.

### LD decay analysis

**Fig. 2:**
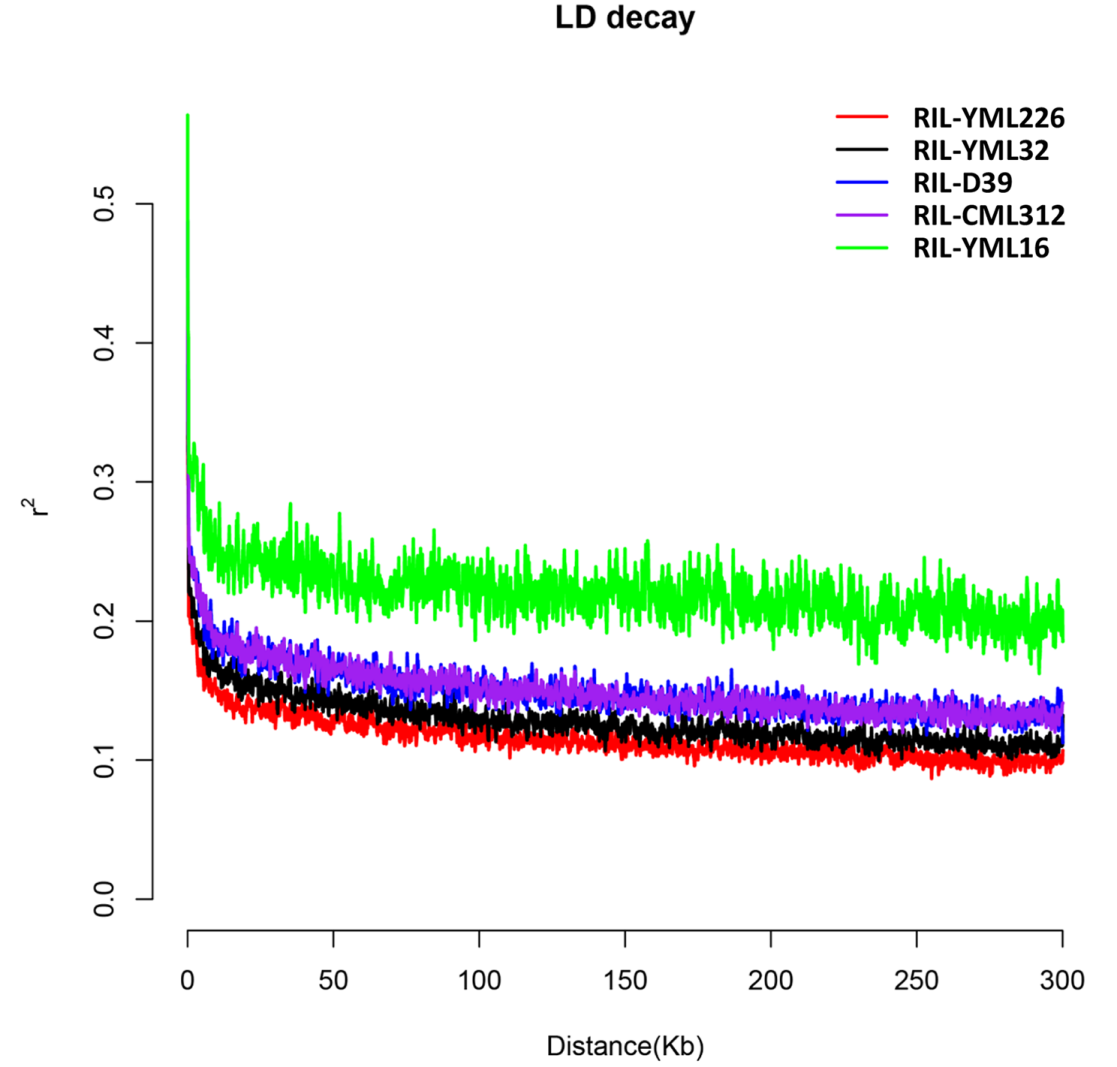
The LD decay of five recombinant inbred line (RIL) populations. LD decay determined by squared correlations of allele frequencies (r^2^) against the distance between polymorphic sites in RIL-YML226 (red), RIL-YML32 (black), RIL-D39 (blue), RIL-CML312 (purple) and RIL-YML16 (green)

Raw SNP dataset from each RIL population was used for linkage disequilibrium (LD) decay analysis. We calculated the LD delay for each population and found that the physical distances were approximately 10 to 20 kb when the rates of r^2^ decrease leveled out (Fig. 2). Meanwhile, we chose 10 kb as the criterion for screening candidate genes, taking into account that the longest repeat element in the maize genome is 10 kb.

### Genome-wide association study

**Fig. 3:**
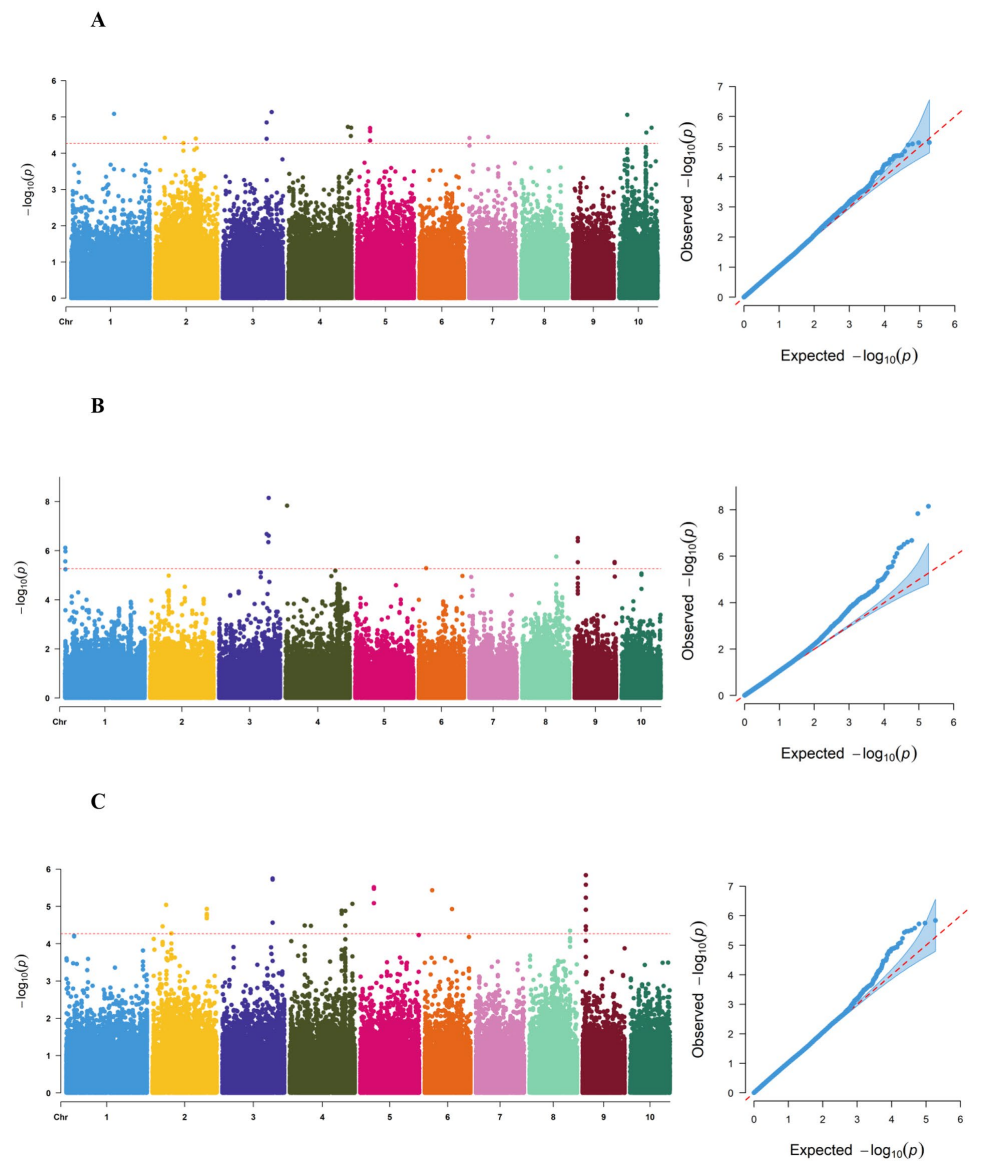
Results of GWAS for GLS resistance. (**A**) Manhattan plot(left) and QQ plot(right) for Dehong (DH) phenotype mean, (**B**) Manhattan plot(left) and QQ plot(right) for Baoshan (BS) phenotype mean, (**C**) Manhattan plot(left) and QQ plot(right) for best linear unbiased estimates (BLUE). The y-axis represents –log10(*p*) values for marker–trait association and the x-axis represents the chromosomes with position. The horizontal red dashed line in the Manhattan plot indicates the significance threshold, and the different colored dots represent the physical location of SNPs on the corresponding chromosomes. The red dashed line in the QQ plot indicates the expected significance value, and the blue dots indicate the actual significance value

GWAS was conducted using the linear mixed model (LMM) in GEMMA, incorporating SNPs and the mean GLS phenotypic data from DH and BS, and as well as the BLUE values. A significant association between SNPs and traits was determined when the *p* < 5.346707e-05 (DH and BLUE) and 5.346707e-06 (BS) (Fig. 3). To address the problem of multiple testing during association analysis, the Bonferroni correction method was applied. The QQ-plot (Fig. 3) indicated the appropriateness of the selected statistical model for the association analysis. Using the phenotypic data from DH, we identified 438 SNPs significantly associated with GLS resistance, which were distributed across chromosomes 1, 2, 3, 4, 5, 7, and 10 of maize. Similarly, using the phenotypic data from BS, we localized 397 SNPs significantly associated with GLS resistance, distributed on chromosomes 1, 3, 4, 6, 8, and 9 of maize. Furthermore, we identified a total of 683 SNPs significantly associated with GLS resistance using the BLUE values, distributed across chromosomes 2, 3, 4, 5, 6, 8, and 9 of maize.

### Candidate genes screening and functional annotation

Based on the significantly associated SNPs obtained from the GWAS, candidate genes were screened within a 10 kb region upstream and downstream of these SNPs. The screening resulted in the identification of a total of 38 genes associated with GLS resistance (Table S1). Among them, thirteen genes were identified at DH location. They were located on chromosomes 2, 3, 4, 5, and 7. Fourteen genes were identified at the BS location and they were distributed on chromosomes 1, 3, 4, 6, and 9. Twenty-one genes were identified through the association analysis using the BLUE values and these genes were distributed on chromosomes 2, 3, 4, 5, 6, and 8. Furthermore, the association analysis revealed that five candidate genes co-localized for the BS location and BLUE values, and five candidate genes were found to be overlapped for the DH location and BLUE values (Table 2).

**Table 2:** Putative candidate genes associated with GLS resistance in maize

Chromosome	Start	End	Candidate genes	Description	Data for GWAS
2	38,091,293	38,098,388	*Zm00001d003257*	*26 S proteasome non-ATPase regulatory subunit 6 homolog*	DH and BLUE
2	38,097,924	38,102,928	*Zm00001d003258*	*Elongator complex protein 3*	DH and BLUE
2	38,105,379	38,121,803	*Zm00001d003259*	*Galactose oxidase/kelch repeat superfamily protein*	DH and BLUE
3	190,965,694	190,973,192	*Zm00001d043188*	*NA-(apurinic or apyrimidinic site) lyase chloroplastic*	DH and BLUE
3	191,414,936	191,416,171	*Zm00001d043199*	*Disease resistance protein RGA4*	BS and BLUE
3	191,416,497	191,430,313	*Zm00001d043200*	*/*	BS and BLUE
5	51,914,095	51,915,783	*Zm00001d014530*	*Phenolic glucoside malonyltransferase 1*	DH and BLUE
6	28,058,154	28,062,105	*Zm00001d035465*	*E3 ubiquitin-protein ligase RHF2A*	BS and BLUE
6	28,062,742	28,064,462	*Zm00001d035466*	*Protein root UVB sensitive 2 chloroplastic*	BS and BLUE
6	28,070,797	28,073,319	*Zm00001d035467*	*Putative CRINKLY4-like receptor protein kinase family protein*	BS and BLUE

### Haplotype-based association analysis

Three adjacent polymorphic SNPs were used as a single haplotype block, and association analysis was performed using the GLS phenotype to identify significant haplotype blocks. The identified significant haplotype blocks were then compared with the candidate genes screened earlier, resulting in the discovery of nine haplotype blocks that overlapped with five candidate genes (Table 3). Among them, haplotype blocks WIN247913 and WIN247914 on chromosome 2 were shared by BS, DH and BLUE, and these two haplotype blocks overlapped with the gene *Zm00001d003257*. Haplotype block WIN457430 on chromosome 3 overlapped with the gene *Zm00001d043188*. Haplotype block WIN1043856 on chromosome 6 overlapped with the gene *Zm00001d035465*. Haplotype blocks WIN1043864, WIN1043865, WIN1043868 and WIN1043869 overlapped with the gene *Zm00001d035466*. Additionally, the haplotype block WIN1043918 on chromosome 6 overlapped with the gene *Zm00001d035467*.

**Table 3:** Haplotype blocks overlapped with the candidate genes†

Candidate gene	Overlapped haplotype blocks	Data for haplotype-based GWAS
*Zm00001d003257*	WIN247913 and WIN247914	BS, DH and BLUE
*Zm00001d003258*	NA	NA
*Zm00001d003259*	NA	NA
*Zm00001d043188*	WIN457430	BS
*Zm00001d043199*	NA	NA
*Zm00001d043200*	NA	NA
*Zm00001d014530*	NA	NA
*Zm00001d035465*	WIN1043856	BS, DH and BLUE
*Zm00001d035466*	WIN1043864/WIN1043865/WIN1043868/WIN1043869	BS, DH and BLUE
*Zm00001d035467*	WIN1043918	BS, DH and BLUE

†NA = not applicable

### Phenotype differences in haplotype blocks

**Fig. 4:**
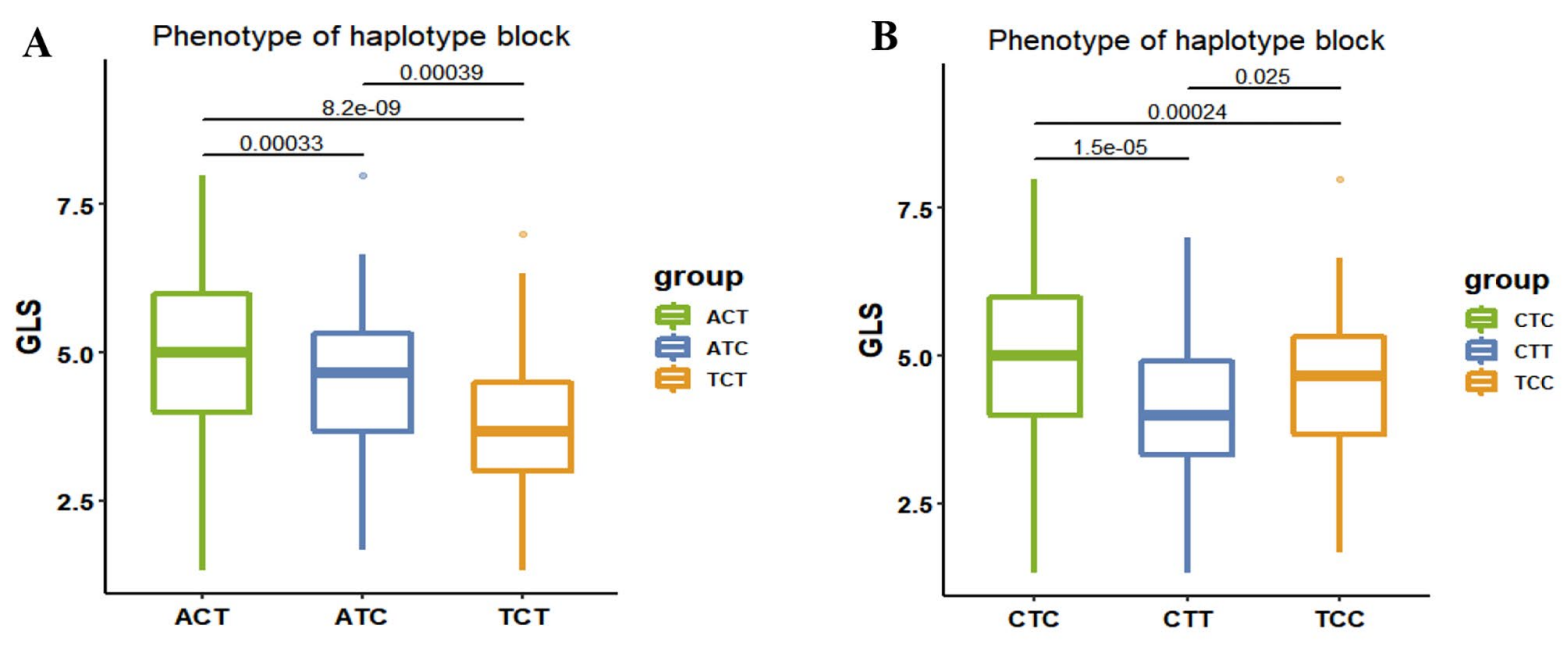
Phenotype differences in haplotype blocks of the gene *Zm00001d003257*. (**A**) Haplotype block WIN247913; (**B**) Haplotype block WIN247914. The vertical axis represents the GLS disease scales. The boxes represent different haplotype block groups. The numbers between any two groups represent *P* values from *t* test

The candidate gene *Zm00001d003257* overlapped with two significant haplotype blocks, viz. WIN247913 and WIN247914. Significant phenotypic differences in plant GLS scales were observed between these two haplotype blocks. Within the WIN247913 block, three haplotype variants were identified: ACT, ATC and TCT (Fig. 4A). All three haplotypes exhibited resistance to GLS based on the corresponding phenotypic data. However, the TCT haplotype block demonstrated the strongest resistance to GLS, with resistance predominantly concentrated around scale 3. Similarly, within the WIN247914 block, three variants were identified: CTC, CTT and TCC (Fig. 4B). All three haplotypes corresponded to phenotypic data indicating resistance to GLS. However, the CTT haplotype block displayed the strongest resistance to GLS, with resistance primarily concentrated around scale 4.

### Expression analysis of candidate genes

**Fig. 5:**
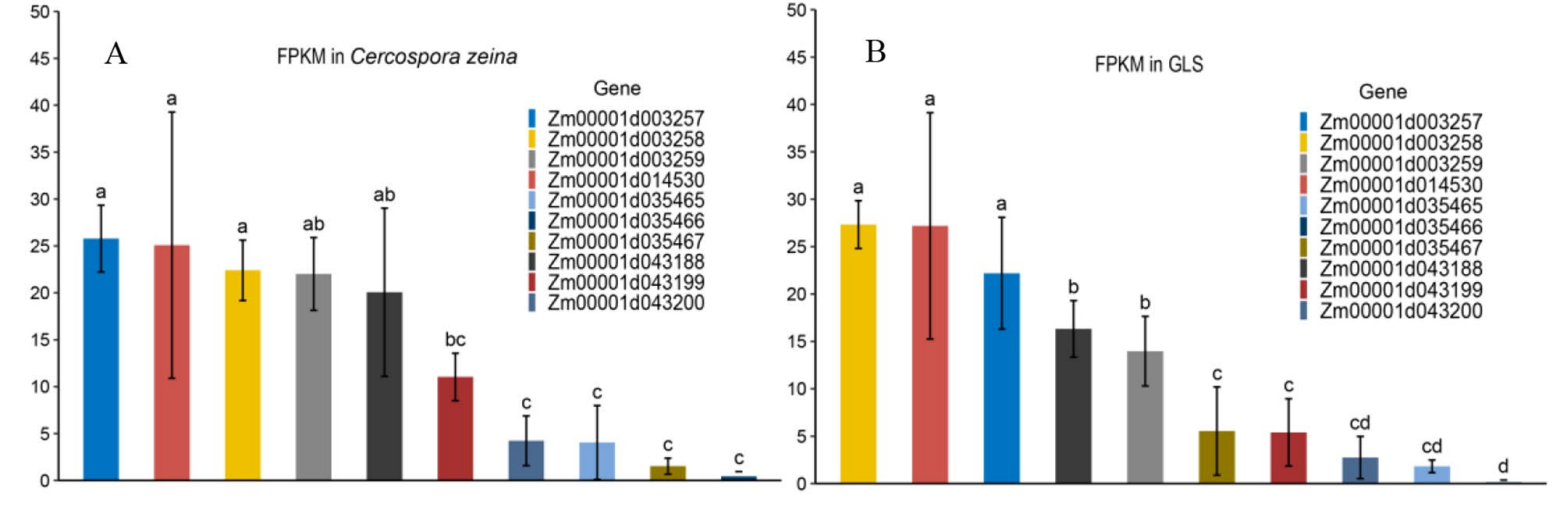
Expression of candidate genes. (**A**) FPKM (Fragment Per Kilobase Million) under GLS stress caused by *Cercospora Zeina*; (**B**) FPKM under GLS stress without specific GLS race identified. The vertical axis represents FPKM. Different colored boxes represent different candidate genes. The *t* test was performed for FPKM of each gene. The bar chart with same letter indicate no significant difference and the black lines represent error bar

Comparing the fragments per kilobase of exon model per million mapped fragments (FPKM) values of the ten candidate genes overlapped with haplotypes shown in Table 3, we found that, except for five genes (*Zm00001d035465*, *Zm0001d035466*, *Zm00001d035467, Zm00001d043199 and Zm00001d043200*), all the other five genes had FPKM values near 20 under *Cercospora zeina* stress (Fig. 5A). Among them, the candidate gene *Zm00001d003257* had FPKM values about 25, and the candidate gene *Zm0001d003258* had FPKM values above 20 under *Cercospora zeina* stress (Fig. 5A). Additionally, all five candidate genes (*Zm00001d003257, Zm00001d003258, Zm00001d003259, Zm00001d014530 and Zm00001d043188*) had FPKM values greater than 10 when the plants were infected with GLS, with the candidate gene *Zm00001d003257* having FPKM values above 20 and the candidate gene *Zm00001d003258* having FPKM values above 25 (Fig. 5B).

## Discussion

The studies on GLS resistance in maize have primarily focused on QTL mapping. Bubeck et al. [15] located QTLs associated with GLS resistance on all 10 maize chromosomes in three F_2:3_ mapping populations, with nearly all markers exhibiting additive action. Balint-Kurti et al. [16] identified five significant QTLs for GLS resistance, including one at bin 2.04 that conferred resistance to southern leaf blight, using an RIL population derived from a cross between the resistant line Mo17 and the susceptible line B73. Zhang et al. [17] detected four QTLs (on chromosomes 1, 2, 5, and 8), designating *qRgls1* and *qRgls2* as major QTLs on chromosomes 8 and 5, respectively. Subsequently, Zhong [18] fine-mapped and validated the *qRgls1* QTL, further identifying and confirming *ZmWAK-RLK* as a GLS resistance gene. In another study, Benson et al. [19] found 16 QTLs employing a nested association mapping (NAM) population, three (*qGLS1.04*, *qGLS2.09* and *qGLS4.05*) of which reduced GLS severity by over 10%. Liu et al. [9] identified seven QTLs associated with GLS resistance, with *qRgls.yaas-8-1*, located within the bin 8.04 interval of chromosome 8, having the highest effect. Chen et al. [20] genotyped two RIL populations, (CML373 × Ye107 and Chang7-2 × Ye107) and discovered 11 QTLs associated with GLS resistance, with individual QTLs explaining 2.05–24.00% of the phenotype variation. Qiu et al. [21] used a near-isogenic line (NIL) population to localize *QTL-qGLS8* for GLS resistance on chromosome 8. Sun et al. [22] identified a QTL, *qRgls1.06*, associated with GLS resistance, explaining 55% of the phenotypic variation using bulked segregant analysis (BSA) and QTL mapping in a backcross population. In our study, three candidate genes, *Zm00001d035465*, *Zm00001d035466* and *Zm00001d035467*, located within the previously reported *QTLGLSchr6* (26909746-120538443kb) [23] region. Moreover the other three candidate genes, *Zm00001d043188*, *Zm00001d043199* and *Zm00001d043200*, were very close to the previously reported significant SNP *qGLS3.07* (196,578,413 kb) [24].

In this study, we conducted SNP-based and haplotype-based GWAS and identified ten candidate genes. Though qRT-PCR is standard and preferred method for validating gene functions, due to some technical problems, the pathogen culture was not success in our lab. Instead we have used FPKM as a way to validate the candidate genes identified by this study. As the result, FPKM analysis allowed us to select five candidate genes with haplotypes showing significant differences in GLS scales from the ten candidate genes. Among these five genes, only *Zm00001d003257* exhibited a haplotype block overlapping with it, and this haplotype block was identified in the haplotype based-GWAS of BS, DH and BLUE. Notably, the TCT haplotype block within *Zm00001d003257* had higher FPKM values compared to other haplotypes under *C. zeina* stress conditions. The *Zm00001d003257* gene encodes the 26 S proteasome regulatory subunit Rpn7/COP9 signaling complex subunit 1. This gene has been shown to upregulate or downregulate gibberellins (GAs) in rice in response to external stresses [25], playing a crucial role in plant growth and development, and immune responses. Both endogenous and exogenous GAs have been found to induce disease resistance and susceptibility in rice against different pathogens [26]. For instance, studies by Nahar et al. [27] revealed that GAs synthesis-impaired, GAs-insensitive, and *SLR1* gain-of-function mutants exhibited enhanced susceptibility, while *SLR1* loss-of-function mutant slr1-1 displayed enhanced disease resistance during rice and *Pythium graminicola* intercropping compared to the wild type. Additionally, Hossain et al. [28] reported that exogenous gibberellins enhanced resistance to *Hirschmanniella oryzae* in rice. Furthermore, exogenous GAs and GAs synthesis inhibitor treatments have been shown to decrease and enhance disease resistance against *Magnaporthe oryzae* and *Xanthomonas oryzae* pv. *oyrzae*, in rice, respectively [29–31]. GAs-insensitive mutants and functionally acquired mutants of *OsSLR1* also exhibited enhanced resistance to rice blast or rice bacterial leaf blight [29, 32]. Based on these previous studies and our findings, we hypothesize that the candidate gene *Zm00001d003257* may possess a similar function in regulating gibberellin content, thereby affecting GLS resistance in maize.

Currently, marker-assisted introgression has been used as a valuable tool in maize breeding. For instance, researchers successfully introduced the β-carotene hydroxylase (crtRB1) gene into low- carotenoids maize varieties, resulting in an approximately 12-fold increase in β-carotene content [33]. In another study, the marker-assisted introgression of qHSR1 improved maize resistance to head smut [34]. Additionally, researchers have introduced maize germplasm lines introgressed with disease resistance genes from teosinte and successfully cloned the major QTL *qLMchr7*, which controls the spot-like phenotype, using map-based cloning. The *ZmMM1* gene, located in qLMchr7, provides maize with broad-spectrum resistance to the disease [35]. The genes we report here may serve as a basis for validating gene functions and prove useful in marker-assisted introgression for maize breeding.

## Conclusion

We identified five candidate genes associated with GLS resistance by employing SNP-based GWAS, haplotype-based GWAS, and expression information from a public RNA database. Combined with previous studies, we further detected one novel candidate gene associated with GLS resistance. The novel candidate genes we identified appeared to be associated with phytohormone regulation or synthesis. Previous studies have shown that phytohormones play a crucial role not only in plant growth and development, but also in plant immune responses. Conducting further functional validation of this candidate gene could offer new insights into GLS resistance mechanisms in maize. Moreover, the genes and loci identified in this study can serve as references for future marker-assisted breeding, and also provide reliable genetic resources for subsequent studies on the mechanisms underlying GLS resistance genes. Importantly, our study also highlights the potential value of tropical or subtropical maize germplasm in breeding maize varieties for GLS resistance.

## Materials and methods

### Experimental materials and field design

Ye107, an important backbone inbred line in China and susceptible to GLS, was used as the common male parent and crossed with four tropical (YML32, YML16, YML226, and D39) and one subtropical (CML312) inbred lines, all of which exhibit stronger resistance to GLS. Through continuous selfing for six generations using the single seed descent method, five RIL populations at the F_7_ generation were obtained. Approximately 200 samples were randomly selected from each RIL population, resulting in the construction of five RIL populations namely, RIL-CML312, RIL-YML32, RIL-YML16, RIL-YML226 and RIL-D39. The parental line names, pedigrees, the heterotic group classification, and their ecotypes are presented in Table 4. The classification of the heterotic groups was based on the “tri-heterotic group” theory, a breeding strategy to improve the selection efficiency of maize hybrids [36]. Initially, each RIL population consisted of 200 samples. However, due to inbreeding depression and other stresses common in inbred line development, few lines were lost during the selfing process. As a result, the final RIL-CML312 population consisted of 151 F_7_ RILs derived from the cross between Ye107 and CML312; the RIL-YML32 population comprised 162 F_7_ RILs derived from the cross between Ye107 and YML32; the RIL-YML16 population with 141 F_7_ RILs derived from the cross between Ye107 and YML16; the RIL-YML226 population with 120 F_7_ RILs obtained from the cross between Ye107 and YML226; and the RIL-D39 population with 183 F_7_ RILs derived from the cross between Ye107 and D39. In total, 757 F_7_ RILs were utilized in this study.

**Table 4:** Information about the parental lines of maize used for developing multi-parent populations

Parents	pedigree	Heterotic group	Eco- types	Symptoms scale of GLS
Ye107	Derived from US hybrid DeKalb XL80	Reid	Temperate	9
CML312	S89500-F2-2-2-1-1-B*5-2-1-6-1(DH)	Non-Reid	Sub-tropical	3
YML32	Suwan 1(S)C9-S8-346-2 (Kei 8902)-3-4-4-6	Suwan	Tropical	1
YML16	GLSIY01HGB-B-27-1-2-B-1-1-2-1(DH)	Non-Reid	Tropical	3
YML226	(CML226/(CATETO DC1276/7619))F2-25-1-B-1-2-1-1-2(DH)	Non-Reid	Tropical	1
D39	Selected from Suwan1	Suwan	Tropical	1

These 757 F_7_ RILs were planted in 2019 at two locations, DH and BS, in Yunnan Province, China. Both locations are known as maize GLS hotspots, experiencing endemic GLS outbreaks almost every year. The experiment was conducted in a randomized complete block design (RCBD) with three replications at each location. Each experimental plot consisted of two 3-m-long rows, with an inter-row spacing of 0.70 m and 14 plants per row. The field trials were conducted according to local standard agronomical practices.

### Phenotyping for GLS and statistical analysis

The RILs of the multi-parent population were screened for GLS under filed conditions at two locations, BS and DH in Yunnan province, China, during the summer of 2019.

The resistance to GLS was assessed starting in the 4th week after maize dispersal. Since the outbreak of maize GLS in Yunnan region occurs in every July when the temperatures range from 20 to 25 °C, with high relative humidity (above 81%), creating conducive conditions for the growth and spread of the GLS spore, five populations were screened during this time to assess the GLS levels. In July 2019, we surveyed GLS in five populations with natural outbreaks of GLS in BS and DH, Yunnan. The scoring criteria for GLS are presented in Table 5 [9, 37], and the GLS resistance score was determined for each RIL population based on the percentage of total leaf area infected by GLS. Descriptive statistical analysis was conducted on the collected data using SPSS software (IBM Corp. Released 2020. IBM SPSS Statistics for Windows, Version 27.0. Armonk, NY: IBM Corp.). Additionally, we employed the lme4 version 1.1–30 [38] R package for calculating BLUE. The one-stage approach was selected, considering location and repetition as random factors, and variety as a fixed factor, to calculate BLUE values. The calculation formula used was: m1 = lmer(GLS ~ Cul + (1|Location) + (1|Location:Rep). The calculated BLUE values, along with the average phenotypic data from BS and DH were used for the subsequent GWAS.

**Table 5:** GLS disease scale used for screening the RILs of Multi-parent populations

GLS scale	Plant reaction to the disease	Area of total leaves
1	No spots on leaves	0–5%
3	A few spots on the lower leaves of the ear	6-10%
5	More spots on the lower leaves of the ear	11-30%
7	Many spots on the lower leaves of the ear and the upper leaves of the ear	31-70%
9	All leaves covered with spots and the leaves wilted	71-100%

### DNA extraction and genotyping-by-sequencing

A genotyping-by-sequencing (GBS) approach was employed to discover SNPs in the 757 maize RILs. Genomic DNA was extracted from seedling leaves of each accession using a modified cetyl trimethyl ammonium bromide method. The modifications included using 4mM tris (2-carboxyethyl) phosphine instead of 2-mercaptoethanol, along with 2% polyvinylpolypyrrolidone and 40 mg RNase [39]. The DNA concentration was assessed using the Quant-iT PicoGreen dsDNA Assay Kit (Life Technologies, Grand Island, NY, United States) and standardized to 20ng/ml for library construction. For the construction of GBS libraries, the methodology described by Poland et al. [40] was followed. Initially, the genomic DNA was digested using PstI and MspI restriction enzymes (New England BioLabs, Ipswich, MA, United States). Subsequently, barcoded adapters were ligated to the digested DNA fragments using T4 ligase (New England BioLabs, Ipswich, MA, United States). Afterward, the ligated products from each plate were pooled and purified using the QIAquick PCR Purification Kit (QIAGEN, Valencia, CA, United States). PCR amplification was then performed using primers complementary to both adaptors. The resulting PCR products underwent additional purification with the QIAquick PCR purification kit and were quantified using the Qubit dsDNA HS Assay Kit (Life Technologies, USA). To ensure the selection of appropriate DNA fragments, size selection was performed using an Egel system (Life Technologies, United States), targeting fragments ranging from 200 to 300 bp. The concentration of each library was estimated using a Qubit 2.0 fluorometer and the Qubit dsDNA HS Assay Kit (Life Technologies, USA). Subsequently, the size-selected libraries were subjected to sequencing using an Ion Proton sequencer (Life Technologies, v 5.10.1) with P1v3 chips after undergoing library preparation on an Ion Chef instrument (Ion PI HiQ Chef Kit). The Ion Torrent system generated sequence reads of varying lengths.

### Quality control and filtering of the raw sequencing reads

Several steps were undertaken in the sequencing workflow to ensure the quality and accuracy of the sequencing data obtained through GBS. For sequencing data processing, FastQC version 0.11.8 was used, and the following criteria and parameters were applied to process the raw reads. Firstly, the adaptor sequence was removed from the samples. Then, reads with bases possessing a quality value of Q ≤ 5, accounting for more than 50% of the entire read, were classified as low-quality reads and removed. Subsequently, the P1 adaptors, each containing 4–8 bp barcode sequences, were added to the reads. This allowed the DNA fragments from different samples to be tagged with distinct barcode sequence, which assist in distinguishing between samples during sequencing. Consequently, the barcode bases were removed from the sequencing reads, allowing the analysis of the selected reads. Finally, reads that couldn’t be differentiated based on the barcode information were discarded. These steps ensured the retention of high-quality reads, which were subsequently used further processing, including sequence alignment and SNP identification.

### Sequence alignment and SNP discovery

The high-quality sequencing reads obtained after quality control were aligned with the maize B73 reference genome (ftp://ensemblgenomes.org/pub/release-40/plants/fasta/zea_mays/dna/Zea_mays.AGPv4.dna.toplevel.fa.gz) using BWA [41]. The alignment was performed with the following parameter: mem -t 4 -k 32 -M. In the resultinh alignment file, we identified and marked highly duplicated SNPs without any deduplication. For SNP detection and extraction, we followed the recommended process outlined in the following resource: (https://gatk.broadinstitute.org/hc/en-us/articles/360036194592-Getting-started-with-GATK4). This process utilized GATK to perform SNP analysis and extraction.

### SNP filtration

Following the SNP calling in the RILs, the quality of each SNP was assessed based on criteria such as minor allele frequency (MAF), the percentage of missing data points, and linkage disequilibrium. Plink v 1.9 [42] was utilized to filter the SNPs, with the parameters set to -geno 0.2 and -maf 0.05, to exclude loci with deletion rates above 10% and loci with minimum allele frequencies below 5%. Initially, the raw molecular marker dataset contained a total of 30,021,334 SNPs, but after filtering 1,730,811 SNPs were retained for GWAS. Additionally, SNP datasets that were filtered based on missing data points, minor allele frequency and linkage disequilibrium were used for population structure analysis.

### Population structure and kinship analysis

#### Principal component analysis

To perform PCA, we used Plink version 1.9 on the filtered SNP datasets. The default parameter values were used, and the top 20 PCAs were retained. The parameter is set to: --pca 20 --threads 10.

#### Ancestral component analysis

Ancestral component analysis provides a more accurate and reliable inference of an individual’s ancestral origin by comparing differences in allele frequencies between populations [43–45]. To achieve this, we utilized Admixture, which employs a maximum likelihood estimation approach to determine individual ancestry based on multi-locus SNP genotype datasets [46]. While Admixture employs the same statistical model as STRUCTURE [47], its fast numerical optimization algorithm allows for faster computational estimation without compromising accuracy compared to STRUCTURE [48]. Therefore, we selected Admixture version 1.3.0 to perform ancestral component analysis on the filtered SNP datasets. The analysis was conducted using default parameters.

#### Construction of phylogenetic tree

We used MEGA version 11.0.13 [49] software to calculate the distance matrix between the 757 RILs based on the filtered SNP dataset. We utilized this distance matrix to construct a phylogenetic tree using the neighbor-joining method. To ensure accuracy, we iteratively calculated the bootstrap values up to 1000 times, while keeping the remaining parameters set as default.

#### Kinship matrix estimation

An unbalanced pedigree among samples is a significant factor contributing to non-linked correlation of markers. The presence of small familial relatedness can lead to false positive in association analysis [50]. Therefore, it is essential to evaluate kinship as a covariate in GWAS. We used GEMMA version 0.98.5 [51] to calculate population kinship matrices for the filtered SNP datasets. The default parameters were employed for the analysis.

### LD decay assessment

Raw SNP data were used to assess LD decay using PopLDdecay v3.42 [52]. The parameters for calculating the r^2^ (correlation coefficient) value were set to their default values. The LD decay figure was drawn with default parameter.

#### Genome-wide association study

GWAS involves assessing genetic variation across the entire genome of multiple individuals to obtain their genotypes. These genotypes are then statistically analyzed in relation to the observed traits, or phenotypes, at the population level. The aim is to identify the genetic variants (markers) that are likely to affect the trait. Statistically significant *p*-values (with Bonferroni correction for calculation) are used to screen the markers, and subsequently, the genes associated with the trait variation are mined [53]. In this study, we used the Linear Mixed Model (LMM) in GEMMA version 0.98.5 [51, 54]. We used PCA and kinship matrix as covariates, and the phenotypes of GLS resistance for GWAS analysis. The calculation formula is as follows:

$$\text{y} = \text{W}{\alpha } + \text{x}{\beta } + {\mu } + {\varepsilon }; {\mu } \sim \text{M}\text{V}\text{N}\text{n}(0, {\lambda }{{\tau }}^{-1}\text{K}), {\varepsilon } \sim \text{M}\text{V}\text{N}\text{n}(0, {{\tau }}^{-1}\text{I}\text{n})$$

In the formula, *y* represents an n-vector of quantitative traits (or binary disease labels) for n individuals; W = (w1,···,wc) is an n × c matrix of covariates (fixed effects) including a column of 1s; α is a c-vector of the corresponding coefficients including the intercept; x is an n-vector of marker genotypes; β denotes the effect size of the marker and is an estimate of the marker/SNP additive effect; ${\mu }$ is an n-vector of random effects; ${\varepsilon }$ is an n-vector of errors; ${{\tau }}^{-1}$ represents the variance of the residual errors; ${\lambda }$ represents the ratio between the two variance components; $\text{K}$ is a known n × n relatedness matrix and In is an n × n identity matrix. $\text{M}\text{V}\text{N}\text{n}$ indicates the n-dimensional multivariate normal distribution.

### Haplotype-based GWAS

For our study, we used Plink version 1.07 to perform association analysis using the filtered SNP data set and the mean phenotype data of GLS resistance collected from BS, DH, and BLUE. The parameters were set to --hap-window 3 --hap-assoc --allow-no-sex --noweb. By applying the same threshold as SNP association analysis, we identified and extracted the significant haplotype blocks.

### Candidate gene expression analysis

We conducted a query on the public database (http://ipf.sustech.edu.cn/pub/zmrna/) to retrieve information related to the expression of the ten candidate genes identified though association analysis. We obtained the FPKM values of the candidate genes under the influence of GLS disease stress.

### Analysis of phenotype differences in haplotype blocks

Based on the chromosome and physical location information of SNPs within significant haplotype blocks, we extracted the corresponding haplotype block information of 757 RILs. This extraction was performed using vcftools v 0.1.16 [55]. We then compared the phenotypic data of these 757 RILs with their respective haplotype blocks, grouping the phenotypic data based on different haplotype blocks.

### Electronic supplementary material

Below is the link to the electronic supplementary material.

Supplementary Material 1

## Data Availability

The datasets presented in this study can be found in online repositories. The names of the repository/repositories and accession number(s) can be found at: https://www.ncbi.nlm.nih.gov/, PRJNA983090. The datasets will be released upon publication of the manuscript.
