# Supplementary material for: Genetic dissection of resistance to gray leaf spot by genome-wide association study in a multi-parent maize population
Source: BMC Plant Biol. 2024 Jan 2;24:10. doi: 10.1186/s12870-023-04701-1 (PMC10759574; doi:10.1186/s12870-023-04701-1)
Supplement: Supplementary file 1 — Supplementary Material 1 [file 12870_2023_4701_MOESM1_ESM.docx]

**Table S1: Genes screened for Significant SNPs associated with GLS resistance in maize.**

| Chromosome | Start | End | Candidate genes |
| --- | --- | --- | --- |
| 1 | 1492439 | 1533158 | *Zm00001d027278* |
| 2 | 38091293 | 38098388 | *Zm00001d003257* |
| 2 | 38097924 | 38102928 | *Zm00001d003258* |
| 2 | 38105379 | 38121803 | *Zm00001d003259* |
| 2 | 206581586 | 206582314 | *Zm00001d006396* |
| 2 | 206585770 | 206587212 | *Zm00001d006397* |
| 2 | 206591023 | 206592594 | *Zm00001d006398* |
| 2 | 206593087 | 206594087 | *Zm00001d006399* |
| 2 | 206594345 | 206597312 | *Zm00001d006400* |
| 2 | 206597858 | 206602521 | *Zm00001d006401* |
| 3 | 183512105 | 183512578 | *Zm00001d042904* |
| 3 | 190146935 | 190151131 | *Zm00001d043147* |
| 3 | 190152574 | 190154270 | *Zm00001d043148* |
| 3 | 190157000 | 190162325 | *Zm00001d043149* |
| 3 | 190965694 | 190973192 | *Zm00001d043188* |
| 3 | 191414936 | 191416171 | *Zm00001d043199* |
| 3 | 191416497 | 191430313 | *Zm00001d043200* |
| 4 | 5628003 | 5632214 | *Zm00001d048792* |
| 4 | 197047799 | 197050973 | *Zm00001d052678* |
| 4 | 232883169 | 232885409 | *Zm00001d053519* |
| 4 | 238064255 | 238068266 | *Zm00001d053640* |
| 4 | 244581797 | 244585737 | *Zm00001d053983* |
| 4 | 244585903 | 244597338 | *Zm00001d053984* |
| 4 | 245438586 | 245441410 | *Zm00001d054037* |
| 5 | 51914095 | 51915783 | *Zm00001d014530* |
| 6 | 28058154 | 28062105 | *Zm00001d035465* |
| 6 | 28062742 | 28064462 | *Zm00001d035466* |
| 6 | 28070797 | 28073319 | *Zm00001d035467* |
| 7 | 729720 | 730607 | *Zm00001d018605* |
| 7 | 734955 | 735896 | *Zm00001d018606* |
| 7 | 741135 | 741789 | *Zm00001d018607* |
| 7 | 745804 | 746844 | *Zm00001d018608* |
| 8 | 154348667 | 154351099 | *Zm00001d011551* |
| 8 | 154352242 | 154353387 | *Zm00001d011552* |
| 8 | 154359742 | 154360633 | *Zm00001d011553* |
| 9 | 153995278 | 153999021 | *Zm00001d048291* |
| 9 | 154002038 | 154006592 | *Zm00001d048292* |
| 9 | 154011332 | 154012566 | *Zm00001d048293* |
